# Identifying and quantifying isoforms from accurate full-length transcriptome sequencing reads with Mandalorion

**DOI:** 10.1186/s13059-023-02999-6

**Published:** 2023-07-17

**Authors:** Roger Volden, Kayla D. Schimke, Ashley Byrne, Danilo Dubocanin, Matthew Adams, Christopher Vollmers

**Affiliations:** 1 Department of Biomolecular Engineering, University of California Santa Cruz, Santa Cruz, CA 95064 USA; 2 Present Address: Pacific Biosciences, Menlo Park, CA 94025 USA; 3 Department of Molecular, Cellular, and Developmental Biology, University of California Santa Cruz, Santa Cruz, CA 95064 USA; 4 Present Address: Genentech, San Francisco, CA 94080 USA

## Abstract

In this manuscript, we introduce and benchmark Mandalorion v4.1 for the identification and quantification of full-length transcriptome sequencing reads. It further improves upon the already strong performance of Mandalorion v3.6 used in the LRGASP consortium challenge. By processing real and simulated data, we show three main features of Mandalorion: first, Mandalorion-based isoform identification has very high precision and maintains high recall even in the absence of any genome annotation. Second, isoform read counts as quantified by Mandalorion show a high correlation with simulated read counts. Third, isoforms identified by Mandalorion closely reflect the full-length transcriptome sequencing data sets they are based on.

**Supplementary Information:**

The online version contains supplementary material available at 10.1186/s13059-023-02999-6.

## Introduction

In any eukaryotic cell, alternative splice site, transcription start site, and polyA site usage shape transcriptomes by enabling the expression of multiple unique isoforms for any one gene [1]. Understanding which isoform is expressed for a gene in a specific sample, be it a single cell or a bulk tissue, is crucial to understanding the biological state of that sample. Consequently, developing tools that gather a complete isoform-level understanding of the transcriptome within a sample is one of the main remaining challenges in genomics. With huge consortium projects such as the Earth BioGenome Project [2] now working on expanding our understanding of genomic diversity across species by sequencing and assembling new genomes, it is particularly important to develop isoform identification tools that do not depend on previously generated and curated genome annotations.

Tools designed to process data generated by the ubiquitous RNA-seq assay fail at the isoform-level analysis of transcriptomes [3–5] because RNA-seq uses short sequencing reads to sequence fragmented transcripts. Inferring or assembling accurate transcript isoforms from fragmented transcript sequences has proven to be extremely challenging [6]. However, while short-read platforms like Illumina are limited to read lengths of a few hundred nucleotides—much shorter than average transcripts, Pacific Biosciences (PacBio) and Oxford Nanopore Technologies (ONT) long-read platforms now routinely generate read lengths of tens of thousands of nucleotides—far longer than average transcripts.

Long-read platforms therefore made it possible to sequence transcripts end-to-end which in turn gave rise to the growing field of full-length transcriptome sequencing assays. To overcome the high error rate inherent to both PacBio and ONT platforms, the PacBio CCS/Iso-Seq method and the ONT-based R2C2 method generate consensus sequences from very long but error-prone raw reads. The millions of highly accurate end-to-end transcript sequences that PacBio Iso-Seq [7, 8] and ONT-based R2C2 [9–11] methods are producing have already been applied to bulk and single-cell transcriptome analysis. These studies have shown that data sets produced by these methods simplify the task of identifying isoforms dramatically, because if single accurate sequencing reads cover entire transcripts, inference or assembly of isoforms as done for fragmented data is unnecessary. Instead, isoforms can simply be defined by grouping and summarizing read alignments based on their features (i.e., alignment starts/ends and splice junctions).

Indeed, new computational tools have been developed or existing tools adapted to take full advantage of this new data type. However, many of these tools rely heavily on previously generated genome annotations. As part of the LRGASP consortium, several isoform identification tools, including StringTie [3, 12], IsoQuant [13], IsoTools [14], Bambu [15], FLAIR [16], FLAMES [17], TALON [18], and our own Mandalorion, were compared by a group of independent evaluators to assess performance [19].

Here, we introduce and benchmark version 4.1 of Mandalorion. Mandalorion v4.1 identifies isoforms with very high recall and precision when applied to either spike-in or simulated data with known ground-truth isoforms. Mandalorion v4.1 outperforms or matches StringTie (v2.2.1), Bambu (v3.08), and IsoQuant (v3.2.0) when identifying and quantifying isoforms with annotation files provided. Importantly, Mandalorion had a distinct performance lead when tools were run entirely without annotation files. Running tools entirely without an annotation allows us to evaluate performance within poorly or incompletely annotated genomes like those of non-model organisms. We also show that Mandalorion not only accurately identifies isoforms but accurately quantifies isoform levels. Finally, by analyzing public PacBio Iso-Seq data, we show that, in contrast to isoforms identified by the other tools, isoforms identified by Mandalorion closely reflect the data set they are based on. Together, this establishes Mandalorion as an excellent choice when analyzing any high-accuracy long-read transcriptome data set in any context.

## Results

### Mandalorion workflow

Mandalorion (v4.1) accepts an arbitrary number of FASTA/Q files containing accurate full-length transcriptome sequencing data. While Mandalorion is generally platform and method agnostic, it is unlikely to work with data sets with error rates higher than 3% and has only been tested on high-accuracy end-to-end transcript sequences which can be generated by either the ONT-based R2C2 method or the PacBio Iso-Seq method—both of which have median error rates lower than 0.5%.

**Fig. 1:**
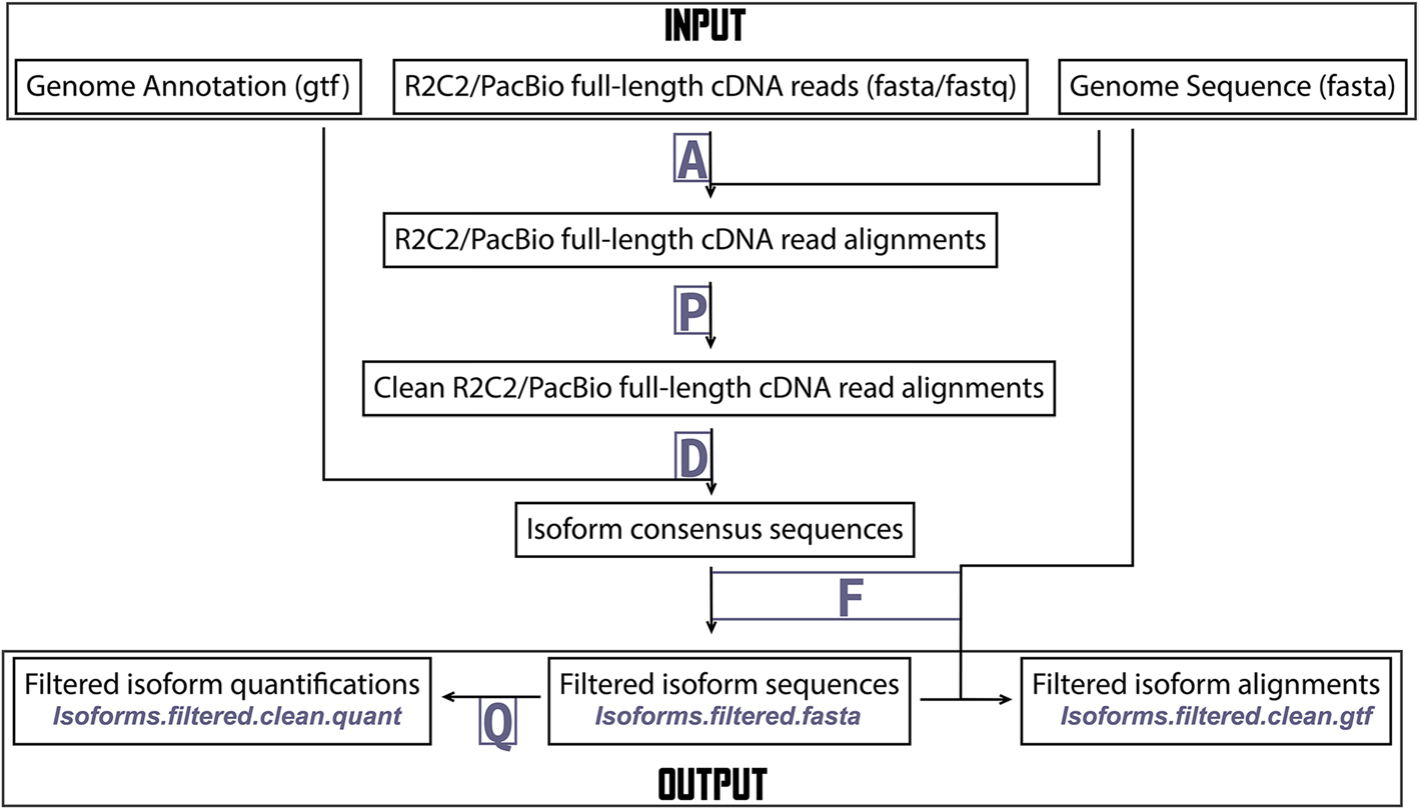
Mandalorion workflow. Input files, processing steps, and output files are shown in a workflow diagram. Using several modules (A, P, D, F, and Q), Mandalorion aligns reads to a genome sequence (using minimap2), groups reads into isoforms based on those alignments, and generates a consensus sequence for each isoform (using pyabpoa). It then aligns these isoform sequences (using minimap2) and filters the isoforms based on those alignments

Mandalorion is organized into several modules (A, P, D, F, and Q) which by default are all run sequentially (Fig. 1). The “A” module aligns reads using minimap2 [20]. Next, the “P” module converts and cleans (removing small indels) the resulting read alignments, and the “D” module then processes the resulting clean read alignments locus by locus to (1) identify high-confidence splice sites, (2) group reads into junction-chains based on the splice sites they contain, (3) identify high-confidence TSS and polyA sites for each junction chain, (4) define (potentially several) isoforms for each junction-chain based on TSS and polyA usage, and (5) generate consensus sequences for each isoform using pyabpoa [21]. The “F” module then aligns and filters the isoform consensus sequences (FASTA) to generate isoform models (GTF and PSL) and groups isoforms into loci associated with annotated genes. Finally, the “Q” module quantifies the filtered isoforms across all provided input FASTA/Q files and groups these isoforms into gene loci.

### Evaluation of Mandalorion

We compared Mandalorion (v4.1), StringTie (v.2.21), Bambu (v3.08), and IsoQuant (v3.2.0) for the identification of isoforms from both simulated and real PacBio reads—whether or not a genome annotation was provided. We focused our comparison on StringTie, Bambu, and IsoQuant because they do not require a genome annotation file as input.

First, we ran these tools on mouse data produced or simulated for the LRGASP consortium and available from ENCODE. Second, we ran the tools on publicly available Universal Human Reference (UHR) PacBio Iso-Seq data.

The simulated mouse data contained a subset of annotated isoforms present in the GENCODE genome annotation as well as artificial isoforms not present in the GENCODE genome annotation provided to the tools. The genome annotation—if provided to the tools—therefore contained many annotated transcripts that were not simulated and did not contain any of the simulated artificial transcripts. To evaluate the performance of each tool on this simulated data set—with or without provided genome annotation—we compared the isoforms they identified to the ground truth annotation—all isoforms that were actually present in the simulated data set (annotated or artificial).

The real mouse PacBio data contained SIRV (set 4, Lexogen) synthetic spike-in transcript isoforms of known sequence. All SIRV isoforms were present in the genome annotation provided to the tools as part of synthetic gene loci. No additional “decoy” isoforms were present in the annotation either, meaning that tools were provided the ground truth for the SIRV isoforms. To evaluate the performance of each tool on these synthetic transcript isoforms—with or without provided genome annotation—we compared the isoforms they identified to the same ground truth annotation.

To compare the isoforms identified by each tool for each data set, we used SQANTI [22] categorization. For ground truth-based analysis, isoforms scored as full_splice-match (FSM) to a ground truth isoform were considered as true positives (TP) but only if their ends matched the ground truth isoform to within 50 nt. This in turn allowed us to calculate recall (TP/(TP + FN)) and precision (TP/(TP + FP)).

For the analysis of the UHR data, which lacks a ground truth, we evaluated how isoforms identified by each tool and condition were categorized by SQANTI, supported by read alignments, and shared between conditions and tools.

To start, we analyzed simulated and real PacBio Iso-seq data with Mandalorion, StringTie, Bambu, and IsoQuant and calculated recall and precision for each tool.

#### Evaluating Mandalorion isoform identification performance with annotation

**Fig. 2:**
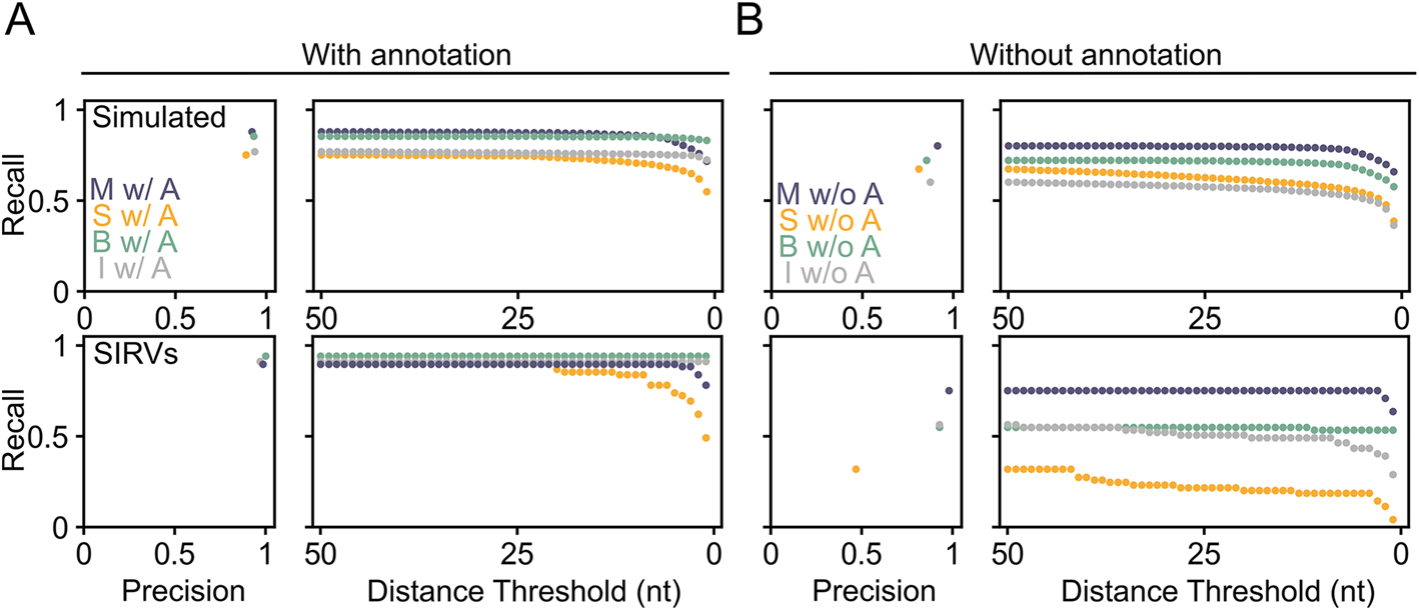
Mandalorion performance is least affected by the absence of annotation. Recall and precision of Mandalorion (M), StringTie (S), Bambu (B), and IsoQuant (I) are shown for simulated and real PacBio data. Recall and precision are shown for tools run with (**A**, “w/A”) or without (**B**, “w/o A”) annotation. Recall is also shown with more stringent distance thresholds applied

With an annotation file available, Mandalorion closely matched all other tools when analyzing simulated or real PacBio Iso-Seq data. When analyzing simulated PacBio Iso-Seq data, Mandalorion reached recall and precision of 87.89% and 92.05% compared to 77.91% and 91.86% reached by StringTie, 85.44% and 92.94% reached by Bambu, and 77.10% and 93.43% reached by IsoQuant, respectively (Additional file 1: Table S1, Fig. 2A). When analyzing SIRV spike-ins in real PacBio data, Mandalorion reached recall and precision of 89.85% and 98.41% compared to 91.30% and 96.92% reached by StringTie, 94.20% and 100% reached by Bambu, and 91.30% and 96.92% reached by IsoQuant, respectively (Additional file 1: Tables S1 and S2, Fig. 2A).

With increasingly stringent distance thresholds, the recall of StringTie declined more sharply than that of Mandalorion, with the recall of Bambu and IsoQuant remaining largely constant (Fig. 2A right).

This shows that, overall, when an annotation file was available, Mandalorion outperformed or closely matched the other tools in both recall and precision for the identification of isoforms from both simulated PacBio reads as well as SIRV spike-ins in real PacBio reads.

#### Evaluating Mandalorion v4.1 isoform identification performance without annotation

Without an annotation file available, Mandalorion outperformed all other tools on recall and precision when analyzing simulated and real PacBio data. When analyzing simulated PacBio Iso-Seq data, Mandalorion reached recall and precision of 80.24% and 91.33% compared to 67.47% and 81.04% reached by StringTie, 72.34% and 85.33% reached by Bambu, and 60.22% and 87.3% reached by IsoQuant, respectively. When analyzing SIRV spike-ins in real PacBio data, Mandalorion reached recall and precision of 75.36% and 98.11% compared to 31.88% and 46.80% reached by StringTie, 55.07% and 92.68% reached by Bambu, and 56.52% and 92.85% reached by IsoQuant, respectively (Additional file 1: Tables S1 and S2, Fig. 2.

Genome browser-style visualizations of the complex loci which the SIRV spike-ins are designed to represent suggest that all tools perform excellently when a genome annotation is provided but, with the exception of Mandalorion, struggle without genome annotation.

**Fig. 3:**
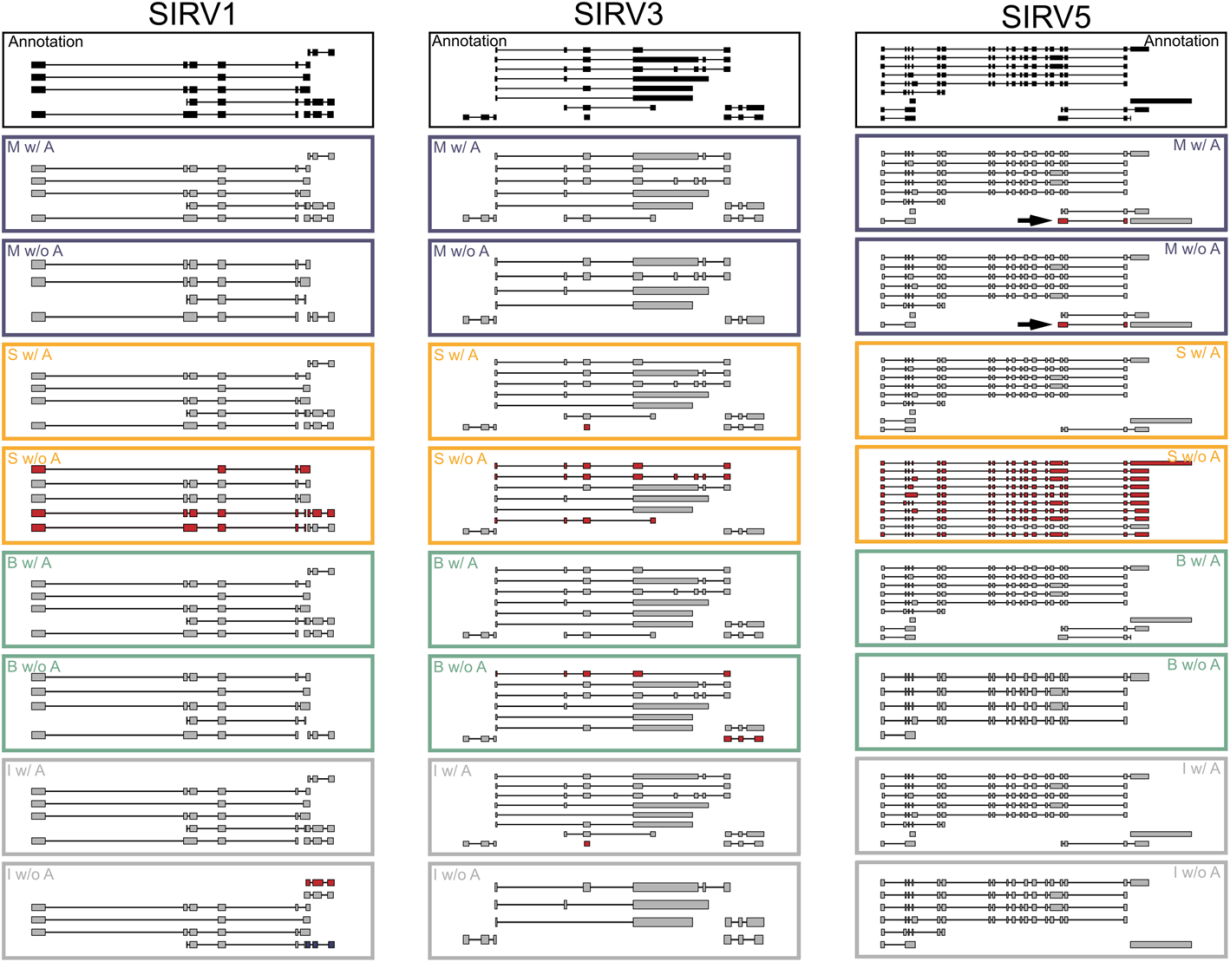
Mandalorion identifies SIRV isoforms consistently with or without genome annotation. Genome browser views of isoforms identified by Mandalorion, StringTie, Bambu, and IsoQuant. True-positive isoforms are shown in gray, and false-positive isoforms are shown in either dark red (unannotated splice junction chain) or dark blue (unannotated ends). Arrows highlight isoform SIRV503

Without genome annotation, StringTie seems to combine the components of multiple separate transcript isoforms into many false-positive isoforms (Fig. 3). Without genome annotation, Bambu and IsoQuant both detect fewer true-positive isoforms for SIRV1, 3, and 5 as well as a few false positives—either with unannotated splice junction chains (SJC) or unannotated ends. In contrast, Mandalorion performs very similarly between conditions. The only false-positive isoform identified by Mandalorion in both conditions was associated with spike-in transcript isoform SIRV503. Interestingly, while the isoform model generated by Mandalorion for this isoform lacked a 7-nucleotide terminal exon, the read-derived consensus sequence generated by Mandalorion for this isoform contained these 7 nucleotides. This highlights that even if a perfect consensus sequence is generated for an isoform, identifying very short terminal exons will continue to represent a formidable challenge for any long-read aligner.

Overall, for both simulated and real PacBio Iso-Seq data, Mandalorion showed excellent isoform identification performance, on par or better than StringTie, Bambu, and IsoQuant when an annotation file was available. When no annotation file was available, Mandalorion clearly outperformed StringTie, Bambu, and IsoQuant on both recall and precision.

#### Evaluating Mandalorion v4.1 isoform quantification performance

In addition to identifying isoforms, Mandalorion also quantifies isoform abundance by counting the number of full-length sequencing reads associated with each isoform. To evaluate and compare the performance of Mandalorion, StringTie, Bambu, and IsoQuant run with annotation, we focused our analysis on 23,882, 20,439, 19,726, and 20,935 isoforms correctly identified by Mandalorion, StringTie, Bambu, and IsoQuant based on simulated PacBio data (SQANTI category FSM, less than 50 nt distance to annotated ends).

**Fig. 4:**
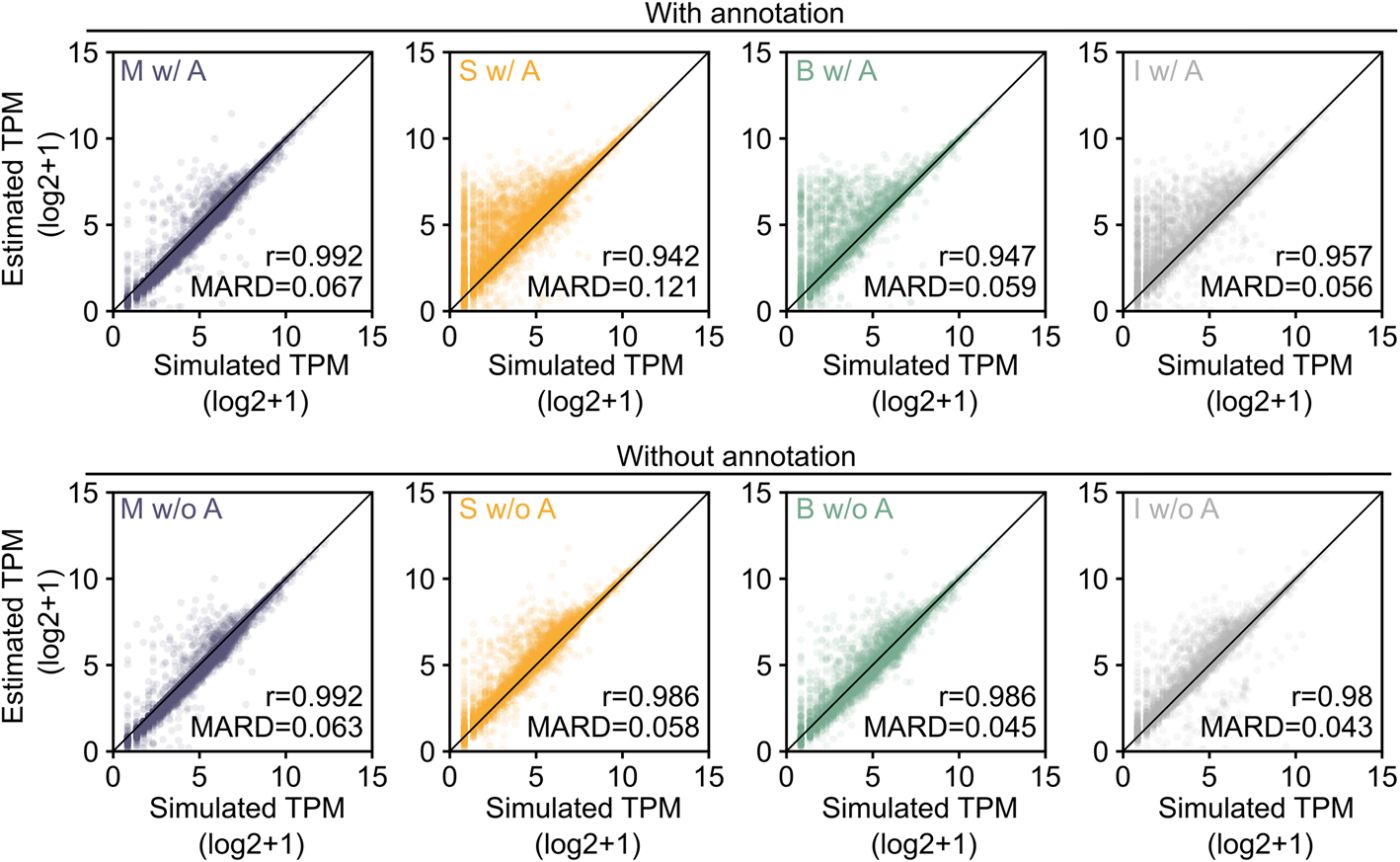
Mandalorion quantifies isoform levels accurately in simulated data. Isoform levels (transcripts per million (TPM)) for simulated isoforms as quantified by Mandalorion (M), StringTie (S), Bambu (B), and IsoQuant (I) are compared to the actual simulated TPM values as scatter plots. Isoform identification and quantification were performed with (w/ A, top) and without (w/o A, bottom) annotation

We compared the normalized read counts (transcripts per million (TPM)) as determined by the tools for these isoforms to the known TPM simulated for these isoforms. We found that Mandalorion TPM values showed the highest correlation with the ground truth, reaching a Pearson’s *r* value of 0.992 compared to ~ 0.95 reached by the other tools (Fig. 4). The mean absolute relative difference (MARD) when comparing TPMs to the ground truth values was 0.067 for Mandalorion, which was similar to the other tools but was likely affected by Mandalorion consistently slightly undercounting expression values.

We then performed the same comparison for the 21,802, 18,320, 19,642, and 16,388 isoforms correctly identified by Mandalorion, StringTie, Bambu, and IsoQuant without annotation. We found that Mandalorion TPM values continued to show the highest correlation to the ground truth, reaching a Pearson’s *r* value of 0.992 compared to the ~ 0.985 reached by the other tools (Fig. 4). Interestingly, in addition to Pearson’s *r* values, MARD of StringTie, Bambu, and IsoQuant improved as well without annotation. This highlights that while using annotations to identify isoforms can improve recall and precision, it seems to distort quantification.

Both with and without annotation, Mandalorion generated TPM values that correlated most closely with the ground truth but did report values that were consistently a little low, likely due to the filtering of read alignments before they are assigned to isoforms and counted.

#### Evaluating Mandalorion v4.1 isoform identification on UHR Iso-Seq data

Finally, we tested how Mandalorion performed when analyzing publicly available Universal Human Reference (UHR) Iso-Seq data. This data set is generated from Universal Human Reference RNA (Agilent), which is composed of the RNA of 10 diverse human cell lines and is therefore highly complex—probably more complex than most tissue or cell line samples. This data set was generated and released by PacBio and contains about 6.7 million full-length cDNA reads.

Because there is no available ground truth for this data set, we focused on how the identified isoforms matched the GENCODE annotation and whether they reflected the actual read alignments they were based on.

We used SQANTI to match isoforms identified by the tools to the GENCODE annotation and found that in contrast to the other tools, Mandalorion identified a mix of annotated and novel isoforms which did not substantially change in number or composition, whether or not an annotation file was available.

**Fig. 5:**
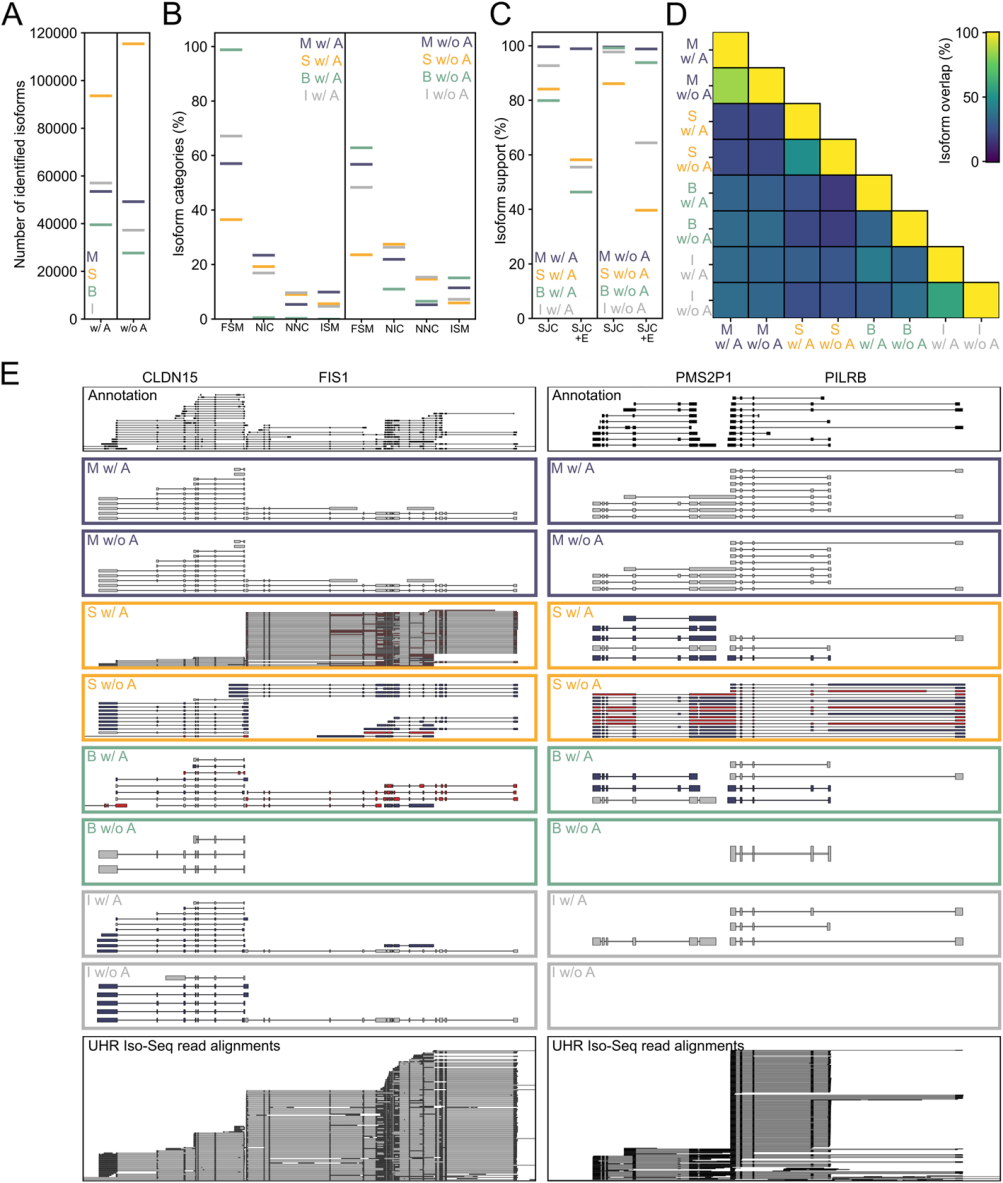
Mandalorion performance is highly stable with or without annotation on the highly complex UHR data set. Number (**A**), SQANTI category composition (**B**), and read support (**C**) of isoforms identified by Mandalorion (M), StringTie (S), Bambu (B), and IsoQuant (I) with (w/ A) or without (w/o A) annotation. FSM = ’full-splice_match’, NIC = ’novel_in_catalog ‘, NNC = ’novel_not_in_catalog’, ISM = ’incomplete-splice_match’. **D** Overlap of isoforms identified by the different tools with or without annotation. **E** Genome Browser views of two genomic loci at chr7:100317083–100369908 (left) and chr7:101229646–101254353 (right). Top to bottom: annotated isoforms; isoforms identified by Mandalorion, StringTie, Bambu, and IsoQuant with (w/ A) or without (w/o A) annotation; and PacBio Iso-Seq reads used for isoform identification are shown. Annotation and Iso-Seq read alignments are shown in black. Identified isoforms with read support are shown in gray. Identified isoforms without read support are shown in dark red (unsupported splice junction chain) or dark blue (unsupported ends)

With an annotation file available, Mandalorion generated 53,493 isoforms of which 57.03% were categorized by SQANTI as FSM, i.e., its entire SJC was present in the GENCODE v38 annotation. StringTie generated 93,611 isoforms of which 36.48% were categorized as FSM. Furthermore, Bambu generated 39,537 isoforms of which 98.78% were categorized as FSM, while IsoQuant generated 57,068 isoforms of which 67.13% were categorized as FSM (Fig. 5A, B).

Without an annotation file available, Mandalorion generated 49,219 isoforms of which 56.76% were categorized as FSM. StringTie generated 115,423 of which 23.57% were categorized as FSM. Furthermore, Bambu generated 27,667 isoforms of which 62.82% were categorized as FSM, while IsoQuant generated 37,276 isoforms of which 48.33% were categorized as FSM (Fig. 5A, B).

Next, we quantified how many of the isoforms identified by each tool were supported by sequence read alignments. We quantified two types of support: (1) “SJC” support, which required at least one read alignment to contain the same SJC as an isoform, and (2) “SJC and isoform ends (SJC + E)” support, which required at least one read alignment to contain the same SJC as an isoform and for the alignment to end within 50 nt of both of the isoform’s ends.

In contrast to the other tools, Mandalorion showed nearly universal read support for the isoforms it identified.

 99.6% and 98.9% of isoforms identified by Mandalorion with annotation showed SJC and SJC + E read support, respectively. In contrast, 80–93% of isoforms identified by StringTie, Bambu, and IsoQuant showed SJC read support, but only 46–58% of these isoforms showed SJC + E support (Fig. 5C). That means that approximately half of the isoforms identified by StringTie, Bambu, and IsoQuant did not have a single read alignment supporting them completely, from end-to-end.

99.6% and 98.8% of isoforms identified by Mandalorion without annotation showed SJC and SJC + E read support, respectively. While read support for isoforms identified with or without annotation was therefore very similar for Mandalorion, support changed strongly for the other tools. SJC support of isoforms increased (86% StringTie, 97.7% IsoQuant, 99% Bambu), while SJC + E support diverged between the tools. 39.7% of StringTie isoforms, 93.9% of Bambu isoforms, and 64.4% of IsoQuant isoforms identified without annotation had SJC + E support (Fig. 5C). Interestingly, that means that if identified without the use of an annotation, isoforms identified by Bambu and IsoQuant are more likely to reflect actual read alignments.

Because of the apparent difference in read support between tools and conditions, we next wanted to investigate the overlap of isoforms identified by Mandalorion, StringTie, Bambu, and IsoQuant. We used GffCompare [23] to group isoforms by their SJCs (but not ends). It is important to note that GffCompare removes isoforms with the same SJC but different ends within each sample before comparison. Based on the gffcompare groups, we then calculated the overlap between pairs of tools and conditions using the Jaccard Index (isoforms shared between pair/(isoforms shared between pair + isoforms unique to sample 1 + isoforms unique to sample 2)) × 100.

By comparing the same tool run with or without annotation available, we found that Mandalorion had the highest overlap with itself at 85.54%, followed by IsoQuant with 58.04% and StringTie at 48.22%. Bambu produced very different isoforms when run with or without annotation with 32.83% overlap. Between tools, the most similar isoform sets were Bambu and IsoQuant run with annotation (41.75% overlap), indicating their mutual heavy reliance on transcript annotations (Fig. 5D).

Overall, this suggests two things. First, in contrast to the other tools we tested, Mandalorion identified a highly similar set of isoforms whether an annotation file is provided or not. Second, Mandalorion isoforms have ends (TSS and polyA sites) that more closely resemble the read alignments they are based on.

We show two examples of this behavior (Fig. 5E) where StringTie, Bambu, and IsoQuant isoforms with/without annotation are entirely different from each other and often not supported (neither SJC nor SJC + E) by any read alignments, while Mandalorion isoforms are highly similar with/without annotation and all supported by read alignments (SJC + E). The example of the PMS2P1/PILRB locus highlights the reliance of tools on annotations exceptionally well. The read alignments to these two genes are highly complex and suggest a potential “read-through” fusion between these two genes which is not annotated. As a result, with the exception of Mandalorion, none of the tools called this fusion when an annotation was provided. Whether or not this fusion is real or an artifact would require follow-up experiments, however, it manifests in multiple isoforms spanning both genes which are all supported by read alignments and therefore should be reported.

Ultimately, this highlights that Mandalorion relies on the fact that the majority of accurate full-length cDNA reads truly cover RNA transcripts end-to-end. We believe this is a strength when analyzing this data type, but it also means that, in contrast to other tools, Mandalorion will identify isoforms that contradict annotations if they are supported by reads and will not identify annotated isoforms unless they are supported by reads, which may not be the case due to molecular biology or sequencing technology limitations for, e.g., very long isoforms.

## Discussion

We initially released Mandalorion in 2017 to identify isoforms based on the then fairly new full-length transcriptome data type [24]. Over the last 6 years, we have continuously developed and used Mandalorion in several publications to analyze bulk and single-cell data sets [9–11, 25, 26]. Version 4.1 is in many ways the culmination of our efforts over the last 6 years to turn Mandalorion from a hard-to-run collection of metaphorically duct-taped together scripts into an easy-to-install and run, robust, fast, and powerful tool. To highlight some improvements over previous versions: Mandalorion v4.1 now (1) requires only two non-standard python libraries (mappy, pyabpoa) and two standalone tools (minimap2, emtrey) which are installed by a setup script, (2) can be run with concise input from the command line (see the “Methods” section), (3) is much faster than previous versions (hours vs. days) and requires less RAM due to optimized multithreading, (4) has much improved error handling and reporting, and (5) has better recall in poorly annotated loci.

Alongside Mandalorion, the full-length transcriptome sequencing field has matured as well and other tools have been designed for isoform identification based on this now-established data type. These tools, which include but are not limited to FLAIR, IsoQuant, IsoTools, TALON, StringTie, Bambu, and FLAMES, present other approaches to the isoform identification problem and their “big-picture” differences can be compared in the LRGASP manuscript [19].

## Conclusions

Here, we perform a separate, distinct analysis to show that Mandalorion represents a strong combination of recall and precision when analyzing PacBio Iso-Seq data—although LRGASP shows that Mandalorion shows the equivalent performance when run on ONT-based R2C2 data or a mix of the two data types.

In our comparison based on publicly available LRGASP and UHR data, Mandalorion compares favorably to StringTie, Bambu, and IsoQuant—especially in the absence of genome annotation. While running a tool entirely without genome annotation does not reflect their likely usage on model organism data, it does allow us to predict performance in poorly annotated gene loci or in any transcriptome/genome combination that lacks a highly curated annotation, i.e., anything that is not human or mouse. Based on its performance here, we make a strong case that Mandalorion is a powerful tool for de novo genome annotation based on full-length transcriptome data.

What sets Mandalorion apart from other tools is how it treats genome annotations (if available), as well as read alignments and their underlying sequences. The only information that Mandalorion extracts from genome annotations is the location of splice sites. It does not collect information about how these sites are connected into splice junctions. It also entirely ignores the transcription start and polyA sites present in the genome annotation. As a consequence of its minimal usage of genome annotation information, Mandalorion is only minimally biased by it. Furthermore, Mandalorion uses alignment quality, specifically around splice sites, to filter reads. We therefore recommend the use of Mandalorion only for high-quality data sets generated by PacBio or ONT-based R2C2. Finally, Mandalorion is unique in generating read-based consensus sequences for each putative isoform which it realigns to define the isoform’s genomic coordinates. This means that Mandalorion is unlikely to report an isoform that is not present in the full-length cDNA data it is processing. To then remove cDNA fragments—the most likely the cause of remaining false-positive isoforms—Mandalorion will discard isoforms that are likely to be internal fragments of other, longer isoforms.

The result of this unique workflow—Mandalorion v4.1—is a strong addition to the toolbox of researchers analyzing full-length transcriptome data. As this data type becomes more common, additional tasks like variant detection and allele-specific isoform analysis will represent new challenges for tools like Mandalorion and represent exciting opportunities for further tool development.

## Methods

### Simulated data

PacBio Iso-Seq data was simulated for the LRGASP consortium effort using IsoSeqSim (https://github.com/yunhaowang/IsoSeqSim). IsoSeqSim simulates truncation and errors at rates estimated using real PacBio cDNA CCS reads. Pre-computed Sequel II truncation probabilities included in IsoSeqSim were used for this purpose. A GTF file containing a subset of GENCODE vM27 and a list of artificial isoforms paired with a file containing abundances for each isoform were used as the underlying isoforms for simulation and served as ground truth for our analysis.

### SIRV PacBio data

PacBio Iso-Seq data of RNA from mouse embryonic stem cell line was generated using an oligodT-priming and template-switching based Smart-Seq2 RT-PCR protocol for the LRGASP consortium effort. The protocol used Maxima H Minus for reverse transcription and SeqAmp for PCR amplification. Before processing, SIRV set 4 was added to the RNA and served as ground truth for our analysis. The resulting libraries were sequenced on the PacBio Sequel II.

### UHR PacBio data

Universal Human Reference RNA (Agilent) was prepped using the Iso-Seq Template Preparation for Sequel Systems (PN 101–070-200) protocol which is also based on the Smart-Seq2 protocol. The resulting libraries were sequenced on the PacBio Sequel II.

### Read preprocessing

#### Simulated PacBio

python3 Mandalorion/utils/removePolyA_nonDirectionalInput.py -i input.fasta -o output.trimmed.fasta -t 1,1

#### Mandalorion

With annotation: python3 Mando.py -p./ -f reads.fofn -W basic,SIRV -G lrgasp_grcm39_sirvs.fasta -t 50 -g lrgasp_gencode_vM27_sirvs.gtf

Without annotation: python3 Mando.py -p./ -f reads.fofn -G lrgasp_grcm39_sirvs.fasta -t 50

#### StringTie

For StringTie we used the alignments generated by running Mandalorion after sorting and converting to bam using samtools [27].

With annotation: stringtie mm2Alignments.sorted.bam -o stringtie_annot.gtf -L -p 50 -G lrgasp_gencode_vM27_sirvs.gtf

Without annotation: stringtie mm2Alignments.sorted.bam -o stringtie_annot.gtf -L -p 50

#### Bambu (in R

library(bambu)

With annotations: bambuAnnotations <—prepareAnnotations(“lrgasp_gencode_vM27_sirvs.gtf”).

se <—bambu(reads = “mm2Alignments.sorted.bam”, annotations = bambuAnnotations, genome = “lrgasp_grcm39_sirvs.fasta”, ncore = 30)

writeBambuOutput(se, path = “bambu/”)

Because Bambu reports newly identified isoforms alongside the entire provided genome annotation in a combined GTF, we parsed this GTF to only keep isoforms if they had a bambu reported read count of at least 3.

Without annotations: se <—bambu(reads = “mm2Alignments.sorted.bam”, annotations = NULL, genome = “lrgasp_grcm39_sirvs.fasta”, ncore = 30, NDR = 1)

writeBambuOutput(se, path = “bambuNoAnnot/”)

#### IsoQuant

With annotation: isoquant.py –genedb isoquant/lrgasp_gencode_vM27_sirvs.gtf –reference lrgasp_grcm39_sirvs.fasta –bam mm2Alignments.sorted.bam –data_type pacbio_ccs -o isoquant/

Without annotation: isoquant.py –reference lrgasp_grcm39_sirvs.fasta –bam mm2Alignments.sorted.bam –data_type pacbio_ccs -o isoquantNoAnnot/

### SQANTI analysis

#### Simulated PacBio LRGASP data

python3 sqanti3_lrgasp.challenge1.py isoform.gtf simulated_isoforms.gtf lrgasp_grcm39_sirvs.fasta –json experiment.json –cage_peak refTSS.mouse.bed –polyA_motif_list polyA_list.txt -c ES_Illumina_STARpass1_SJ.out.tab -d./SQANTI -o output –gtf

#### PacBio LRGASP data

python3 sqanti3_lrgasp.challenge1.py isoform.gtf lrgasp_gencode_vM27_sirvs.gtf lrgasp_grcm39_sirvs.fasta –json experiment.json –cage_peak refTSS.mouse.bed –polyA_motif_list polyA_list.txt -c ES_Illumina_STARpass1_SJ.out.tab -d./SQANTI -o output –gtf

#### PacBio UHR data

python3 sqanti3_lrgasp.challenge1.py isoform.gtf lrgasp_gencode_v38_sirvs.gtf lrgasp_grch38_sirvs.fasta –json experiment.json –cage_peak refTSS.human.bed –polyA_motif_list polyA_list.txt -c WTC11_Illumina_STARpass1_SJ.out.tab -d./SQANTI -o output –gtf

Samtools [27], NumPy [28, 29], SciPy [30], and Matplotlib [31] libraries were used extensively for analysis.

## Supplementary Information

**Additional file 1: Table S1.** Recall and precision of Mandalorion and StringTie versions. **Table S2.** (SIRV tool assignment).

**Additional file 2.** Review history.

## Data Availability

Part of the data used in this manuscript was generated for the LRGASP consortium by several labs. All these data are publicly available. PacBio data for the Mouse F121-9 cell line can be found at ENCODE under file IDs ENCFF874VSI, ENCFF667VXS, and ENCFF313VYZ [32]. Simulated mouse data are available as part of the data provided by the LRGASP consortium at Synapse under ID syn25683381 [33]. PacBio Iso-Seq UHR data are available at https://downloads.pacbcloud.com/public/dataset/UHR_IsoSeq/ [34]. Mandalorion is available at https://github.com/christopher-vollmers/Mandalorion [35] under the MIT license. The exact version used in the manuscript is available from Zenodo at https://doi.org/10.5281/zenodo.7998524 [36].
