# Supplementary material for: Identifying and quantifying isoforms from accurate full-length transcriptome sequencing reads with Mandalorion
Source: Genome Biol. 2023 Jul 17;24:167. doi: 10.1186/s13059-023-02999-6 (PMC10351160; doi:10.1186/s13059-023-02999-6)
Supplement: Supplementary file 2 — Additional file 2. Review history. [file 13059_2023_2999_MOESM2_ESM.docx]

**1^st^ round**

**Reviewer 1**

The paper presents an updated and more usable tool for inferring transcripts and quantification from long read data. I appreciate the objective and the overall study design, including the use of multiple distinct types of data to assess Mandalorion v4's performance (simulated, spike-in, and 'real' data).

I was able to run Mandalorion v4 successfully on a real dataset pulled from the SRA (SRR13422603). I further tried it on simplistic simulated data of full-length transcripts with 3% read errors thrown on top. Running without any provided annotation, Mandalorion gave isoforms to about 15k out of the 20k reads and those isoform annotations matched the true annotation to 99% base overlap in 91% of the reads, confirming Mandalorion's good performance.

A couple of fixable issues could make the paper significantly stronger.

Major comments:

1. Mandalorion is compared to StringTie only. The justification given for this is that StringTie is the only alternative that accepts annotation files. Given the number of alternatives cited by the authors, I believe it is necessary to include more alternatives in the comparisons that do not use annotations. Any alternatives that are not included in the comparison should be justified, for example if they are known to perform worse than other included alternatives based off LRGASP or other prior benchmarks. Without this comparison, it's hard to assess whether Mandalorion represents a improvement over existing pipelines.

2. Description of the simulated data and benchmark is lacking in this paper which makes it hard to understand without reading the citations. In particular, simulated data contains 'spike-in' unannotated transcripts derived from other species, which should be mentioned in text. Are the benchmark TP/FP rates calculated just on those novel spike-in transcripts? Is the annotation-free benchmark looking at all transcripts or just the novel ones?

3. While the use of Universal Human Reference for one benchmark does complement the other benchmark datasets, it is limited by the design (UHR is a combination of 10 human cancer-derived cell lines). It is unlikely that this represents realistic data from any actual experiment, both in transcriptome and in genome. I encourage the use of more realistic samples despite the popularity of the UHR. All of the benchmarks performed in this paper on the UHR could instead be done another, more typical sequencing data. If the authors stick with UHR, it would be good to discuss these limitations.

Minor Comments:

1. The authors could expand on what Mandalorion does differently than other pipelines. I would appreciate some explanation for how it achieves its performance compared to the alternatives. Is Mandalorion's architecture unique or does it make incremental (but important) improvements on prior methods? Why might the other methods perform less well?

2. Fig 3 - could also show false negatives in a third color

3. Fig 3 - center panel: why is the red isoform a false positive? It seems to match with annotation

4. Please check StringTie version number formatting which is inconsistent in a couple places

5. Fig 4 - TPM correlation to truth is good but could also compute other measures of absolute distance to truth, for example the mean absolute relative differences (MARD) like used in Salmon paper (Patro et al 2017, Nature Methods)

6. Fig 5c - A very useful illustration! While this may beyond the scope of the article, it raises the questions of whether the two genes (on the left column) are being transcribed as a unit? Both are well-annotated proteins. Alternatively, this may represent an artefact of the UHR sample, which has an unusually complex genome that may not match the annotation well.

7. What does Mandalorion do with pre-mRNA? Do very long intron-containing pre-mRNA interfere with it's transcript inference?

8. We found that running Mandalorion on simulated data with 10% read error rate to not work (very few isoforms are identified). We chose this error rate as being lower than the values that Minimap2 recommends (up to 15%). Checking the benchmark data the authors used, I found much lower error rates and reran on 3% error rates as stated above (which did work). It would be very helpful for the article to give additional guidance on what kinds of sequencing error rates are acceptable.

9. How does depth of expression affect quantification errors? Is Mandalorion's 3 reads threshold enough to accurately align transcripts (or how much better would 10 be)?

Suggestions of how to improve the experience of using Mandalorion:

1. Installation: need to pip install numpy first. Generally best to specify all dependencies with version numbers in requirements.txt or similar, as well as minimum python version. Ideally, it would be possible to install Mandalorion and the python dependencies via just running: pip install git+https://github.com/christopher-vollmers/Mandalorion

2. pip install pyabpoa also currently fails due to an error in pyabpoa, so maybe best to instruct users to first pip install cython so that it can succeed. (This may be fixed by the above suggestion.)

3. Consider allowing gzipped annotation files since all other input files support gzip

4. If errors are encountered, the pipeline does not abort but instead tries to execute the next command, making it hard to determine where a mistake happened.

5. Minimap2 supports indexes - does Mandalorion allow that and would there be any advantage (say, when aligning many files to the same genome)?

6. Aggregating transcripts into genes is not done, but could be useful (though we would understand if this is challenging and out-of-scope for the current work).

7. When an annotation is provided, it could be very convenient to annotate the inferred transcripts to genes/transcript IDs from the provided annotation. Particularly for novel transcripts of an existing gene (again, possibly out-of-scope)

8. The output file confusingly lists 'hg38' regardless of the genome used

9. Currently, the main difficulty towards using Mandalorion is the multi-step installation process. This also limits repeatability. Packaging it as a Docker image or similar would be convenient for reproducibility and distribution, allowing the inclusion of the dependencies and a single-step installation.

10. Missing files in the fofn input appear to be skipped over but should produce an error and stop

**Reviewer 2**

===
This box is for your report. All comments included here will be passed to the authors.
===
Please consider the following questions:
- Are the methods appropriate to the aims of the study, are they well described, and are necessary controls included?
- Are the conclusions adequately supported by the data shown?
- Are sufficient details provided to allow replication and comparison with related analyses that may have been performed?
- Is the method likely to be of broad utility? Is any software component easy to install and use? Please indicate briefly the novel features and/or advantages of the method, and/or please reference the relevant publications and which methods, if any, it should be compared with.
- Is the paper of broad interest to others in the field, or of outstanding interest to a broad audience of biologists?

Long read based transcriptome sequencing is now considered as state of the art due to the advantages it offers in full length cDNA/RNA sequence generation and isoform detection. Many tools have been developed in the last 4-5 years to enable the analysis of long read data, however the field is still seeking a tool that will take full advantage of this technological advance.

In this method paper the authors present Mandalorion v4, which is the latest development of their isoform detection tool. They measure the performance of v4 using benchmark data (generated by PacBio, the LRGASP consortium and publicly available data), simulated data, as well as through the use of spike-in internal RNA variant controls (SIRVs). Including the SIRV isoform mix is important to provide an experimental benchmark. An important part of the experimental plan included the analysis with and without an annotation file. This is particularly important in the cases where the transcriptomes of non-model, not well annotated organisms are being studied. Transcripts were also quantified and the performance of Mandalorion v4 was compared to the StringTie tool, a widely used isoform detection tool, initially developed for short read data.

Overall, the experimental design utilizes verifiable benchmark data and reagents, and the methodology is well described.

The initial evaluation in terms of recall and precision tests (with and without annotation) was performed using simulated PacBio iso-seq data and SIRVs which provide a truth dataset. The authors claim comparable performance when the annotation file was used, but a superior performance by v4 compared to StringTie, particularly over complex SIRVs, when the annotation file was not used. Quantification performance was similar between the two tools, with generated transcript TPM values highly correlating to the truth set.

Next the authors assessed the performance of the two tools by analyzing a publicly available reference dataset (also PacBio iso-seq based). Here in the absence of ground truth, the authors focused on whether isoforms matched the GENCODE annotation and whether they reflected the actual read alignments they were based on. Again, the performance of Mandalorion v4 was superior when an annotation file was not provided. Importantly, there was concordance between the isoforms detected with and without annotation.

Overall, the development and benchmarking of the fully packaged Mandalorion v4 tool is a welcome addition to the toolkits required to make full use of long read sequencing approaches.
It shows good performance without an annotation file, is of high interest to the transcriptomics and genome annotation community, - and in the view of this reviewer not only for the analysis of not well annotated genomes, but also in the cases of highly complex cancer transcriptomes.

The analysis toll is now well packaged and more user friendly and should allow broad use by researchers

Major comments

1. The authors need to assess the effect of non-full length (originally degraded RNAs, or incomplete cDNAs synthesized) and cDNA synthesis artifacts on the performance of Mandalorion v4. It is expected that real-world data will contain various proportions of such sequences and this issue could be a limiting factor for the usefulness of the tool.
2. The authors base their comparison on the StringTie tool, however the LIQA tool (Hu et al 2021 in this journal) has claimed a higher accuracy than Mandalorion. It is important to include a comparison with this tool, so that readers can obtain a more comprehensive picture of comparative performance.

Minor comments

Although data on isoform read counts are presented (Fig.5B) it is not clear what the read count threshold is (one read?) for calling an isoform and the proportional effect of isoform read counts on recall and accuracy. This needs to be clearly described in the text, as well as a table on saturation, showing raw read numbers required to call the isoforms.

Which version of the SIRVs was used? One can assume that it is set 4, please include in main text.

Figure 2: in the distance threshold graphs it is hard to distinguish between datasets and tools used, the lines run to close. Maybe use different colour scheme/or thinner lines.

Figure 3: it is hard to work out what is not correct with the false positive isoforms when the content of the box is dense. What would be helpful is to clearly show which SIRV isoforms were detected, and which ones were not detected (some seem to escape) by the two tools.

Figure 4: please comment in the text on what appears to be an overestimation of TPMs by the tools and why this would be the case.

Fig 5: Please include the genomic coordinates shown for the annotated loci.

**Authors’ response**

Reviewer #1

1. Mandalorion is compared to StringTie only. The justification given for this is that StringTie is the only alternative that accepts annotation files. Given the number of alternatives cited by the authors, I believe it is necessary to include more alternatives in the comparisons that do not use annotations. Any alternatives that are not included in the comparison should be justified, for example if they are known to perform worse than other included alternatives based off LRGASP or other prior benchmarks. Without this comparison, it's hard to assess whether Mandalorion represents a improvement over existing pipelines.

This is a good point. Because we didn’t want to run tools “off-label” which could cause bad performance, we went through documentation of TALON, FLAIR, IsoQuant, IsoTools, FLAMES, and Bambu. Bambu and IsoQuant now also can be run without an annotation file. We went ahead and ran all the comparisons in this paper with Bambu and IsoQuant as well. This ultimately resulted in significant changes of the entire manuscript but the main conclusion, i.e. Mandalorion performs exceptionally well, and better than the other tools tested, when run without an annotation available, is unchanged.

2. Description of the simulated data and benchmark is lacking in this paper which makes it hard to understand without reading the citations. In particular, simulated data contains 'spike-in' unannotated transcripts derived from other species, which should be mentioned in text. Are the benchmark TP/FP rates calculated just on those novel spike- in transcripts? Is the annotation-free benchmark looking at all transcripts or just the novel ones?

All isoforms that were simulated served as True Positives, not just spike-in transcripts. Our goal here was to run the tools entirely without annotation and seeing how well they do on an entire transcriptome (not just SIRVs). The simulated data serves that purpose very well. We have added text in the method section on data generation and simulation.

3. While the use of Universal Human Reference for one benchmark does complement the other benchmark datasets, it is limited by the design (UHR is a combination of 10 human cancer-derived cell lines). It is unlikely that this represents realistic data from any actual experiment, both in transcriptome and in genome. I encourage the use of more realistic samples despite the popularity of the UHR. All of the benchmarks performed in this paper on the UHR could instead be done another, more typical sequencing data. If the authors stick with UHR, it would be good to discuss these limitations.

This is a good point. As part of the LRGASP paper, Mandalorion was benchmarked on a human cell line (WTC-11), a mouse ES cell line, and a synthetic mix of a human ES cell line and a cell line differentiated from it. It performed very similarly in all conditions there.

We picked UHR because it is a publicly available dataset that of comparable read depth to the LRGASP data but was not part of the LRGASP evaluation. This allowed us to highlight features of Mandalorion while avoiding a significant overlap between this companion paper and the main LRGASP paper.

We have now added in the text how UHR is, as a mix of cell lines, likely highly complex.

Minor Comments:

1. The authors could expand on what Mandalorion does differently than other pipelines. I would appreciate some explanation for how it achieves its performance compared to the alternatives. Is Mandalorion's architecture unique or does it make incremental (but important) improvements on prior methods? Why might the other methods perform less well?

We have now detailed in the discussion why we think Mandalorion performs better than other tools, in particular without a genome annotation. In short, Mandalorion uses provided genome annotations only to white-list splice sites. It does not extract isoform or even splice junction data from the annotations. Because of this, performance with or without annotations is very similar for Mandalorion. Further, it generates consensus sequences for each isoform which it realigns to the genome to create isoform models. This ensures a low False Positive rate.

2. Fig 3 - could also show false negatives in a third color

We tried to implement this but ran into a bunch of issues. With the actual implementation including several edge cases like do you add the full isoform if a partial match is present and so on. We have added a third color however to distinguish unannotated splice-junction chains from unannotated ends to give this figure more nuance and have added a table (S2) that contains all information on the SIRV analysis in detail.

3. Fig 3 - center panel: why is the red isoform a false positive? It seems to match with annotation

We use SQANTI to match identified with annotated isoforms. So if an isoform is shown in red, it doesn’t match an annotated isoform, even if it’s not immediately obvious at the zoomed out view a genome browser view provides. There is however one exception in the form of very short single exon transcripts that are internal and antisense to a larger gene. SQANTI seems to misclassify those in some cases. We now point this out in the text.

4. Please check StringTie version number formatting which is inconsistent in a couple places

We have omitted version numbers now that we are comparing multiple tools and have also dropped Mandalorion v3.6 which at this point is almost a year out of date.

5. Fig 4 - TPM correlation to truth is good but could also compute other measures of absolute distance to truth, for example the mean absolute relative differences (MARD) like used in Salmon paper (Patro et al 2017, Nature Methods)

We have now included MARD values for our comparison. Mandalorion does well here but not always best, likely because it tends to underestimate TPM values.

6. Fig 5c - A very useful illustration! While this may beyond the scope of the article, it raises the questions of whether the two genes (on the left column) are being transcribed as a unit? Both are well-annotated proteins. Alternatively, this may represent an artefact of the UHR sample, which has an unusually complex genome that may not match the annotation well.

We generally see read-through fusion genes quite often for close genes on the same strand in a variety of samples but that’s obviously just anecdotal. We believe these are much more likely to be transcribed as a unit as opposed to being PCR artefact. In any case, we have added a few sentences discussing this case in more detail 7. What does Mandalorion do with pre-mRNA? Do very long intron-containing pre- mRNA interfere with it's transcript inference?

Mandalorion itself handles long unspliced isoforms well as we have observed with the very long unspliced SIRVs which are analyzed for the LRGASP (but not this) manuscript. As long as there is a single splice junction, they are treated as any other isoform. If not, they will be treated as unspliced isoforms, which Mandalorion groups entirely based on isoform ends. In either case, Mandalorion should capture the isoforms as they are present in the cDNA pool that was sequenced.

This is however where the bottleneck for pre-mRNA lies. If pre-mRNA isn’t poly- adenylated yet, it won’t be reverse transcribed with an oligo-dT primer which is currently shared between all full-length cDNA amplification protocols. Even if poly- adenylated, the RT-PCR process used to amplify cDNA is only effective up to about 8kb and can’t really get beyond 12kb at this point. The inclusion of even a single regular human or mouse intron would likely push transcript length beyond that. This is a very long way of saying:

Mandalorion could deal with pre-mRNA but current molecular approaches don’t really capture unspliced transcripts effectively.

8. We found that running Mandalorion on simulated data with 10% read error rate to not work (very few isoforms are identified). We chose this error rate as being lower than the values that Minimap2 recommends (up to 15%). Checking the benchmark data the authors used, I found much lower error rates and reran on 3% error rates as stated above (which did work). It would be very helpful for the article to give additional guidance on what kinds of sequencing error rates are acceptable.

This is expected since Mandalorion discards any read alignment with an identity of less than 90% as pretty much the first step of data parsing. Data of quality this low is very hard to analyze which is why we published the R2C2 method in the first place in 2018.

Mandalorion is intended for the analysis of accurate cDNA reads as represented by R2C2 or Iso-Seq data. Even though we are not developing or testing Mandalorion for this data type, even regular ONT data has an error rate of only 1.5-3% these days. We have added text indicating:

“While Mandalorion is generally platform and method agnostic, it has only been tested on high-accuracy end-to-end transcript molecule cDNA sequences which can be generated by either the ONT-based R2C2 method or the PacBio Iso-Seq method. Because of how Mandalorion filters reads, it is unlikely to work with datasets with error rates higher than 3%.”

9. How does depth of expression affect quantification errors? Is Mandalorion's 3 reads threshold enough to accurately align transcripts (or how much better would 10 be)?

The quantification module of Mandalorion simply counts how many reads are assigned to an isoform. As with any counting based approach, lower counts will be noisier due to counting statistics. When we allow only isoforms with >10 reads to be included in our analysis, r value rises from 0.992 to 0.993 but that improvement wouldn’t really be worth not including the isoforms in our opinion.

Suggestions of how to improve the experience of using Mandalorion:

1. Installation: need to pip install numpy first. Generally best to specify all dependencies with version numbers in requirements.txt or similar, as well as minimum python version. Ideally, it would be possible to install Mandalorion and the python dependencies via just running: pip install git+https://github.com/christopher- vollmers/Mandalorion

We don’t disagree with this but are limited here by the fact that the emtrey sam to psl converter is written in go (which seemed like a good idea at the time). Many linux machines do not have a go compiler installed by default and needs root access to do so.

To at least make the installation easier, we are now including a setup script that installs emtrey and minimap2 locally inside the Mandalorion folder and runs the pip installs for all the required python libraries, including Cython and pyabpoa. We plan on reimplementing emtrey functionality as a function within Mandalorion but this is still a work in progress

2. pip install pyabpoa also currently fails due to an error in pyabpoa, so maybe best to instruct users to first pip install cython so that it can succeed. (This may be fixed by the above suggestion.)

See above

3. Consider allowing gzipped annotation files since all other input files support gzip

We have now implemented this in Mandalorion 4.1

4. If errors are encountered, the pipeline does not abort but instead tries to execute the next command, making it hard to determine where a mistake happened.

Mandalorion will now provide messages when files are missing or empty and then skip ahead to the end of the module. In this way, tracking down errors should be easier

5. Minimap2 supports indexes - does Mandalorion allow that and would there be any advantage (say, when aligning many files to the same genome)?

That’s a good point. We definitely need the fasta file because Mandalorion also scans polyA sites for A content. As for speed, even with a lot of input fasta files, we only run minimap2 once, after concatenating all the fasta files. We simply keep track of what file each read came from. We implemented this after running into an issue when, as part of a single-cell experiment, we had too many fasta files for minimap2 to handle. Overall, I don’t think we’d see a noticeable speed up since for a single run, building an index takes a few minutes but aligning 10 million reads takes a few hours.

6. Aggregating transcripts into genes is not done, but could be useful (though we would understand if this is challenging and out-of-scope for the current work).

We implemented that now as part of the quantifying workflow. Mandalorion now provides a Isoforms.filtered.clean.genes the groups isoforms in loci and associates those with genes if an annotation is provided.

7. When an annotation is provided, it could be very convenient to annotate the inferred transcripts to genes/transcript IDs from the provided annotation. Particularly for novel transcripts of an existing gene (again, possibly out-of-scope)

As noted above, we have now done this for genes. For transcripts, this assignment is more tricky and somewhat subjective, i.e. transcript ends. In this case, we think relying on the excellent GffCompare and SQANTI tools would be a better idea.

8. The output file confusingly lists 'hg38' regardless of the genome used

Yes, that was a placeholder in the PSL to GTF conversion function we wrote and never removed. It should now simply list “Mandalorion” as the source of the isoform.

9. Currently, the main difficulty towards using Mandalorion is the multi-step installation process. This also limits repeatability. Packaging it as a Docker image or similar would be convenient for reproducibility and distribution, allowing the inclusion of the dependencies and a single-step installation.

I agree that a docker would be useful for the group of scientists that are familiar with docker. I personally dislike docker because it adds another tool to the list of things non- computer scientist biologists have to be familiar with to run Mandalorion.

Simplifying installation is definitely something that we should prioritize. That’s why we added a setup script (as mentioned above) that installs all pip installable dependencies (Cython, NumPy, mappy and pyabpoa) as well as a copy of minimap2 and the sam2psl converter emtrey. The only dependencies that this doesn’t cover are go and C compilers which can’t be installed without root access.

10. Missing files in the fofn input appear to be skipped over but should produce an error and stop

This is happening because we use the mappy python library to read FASTA/Q files which errors out quietly but gracefully. As part of our improved error handling, we now check whether input files exist and aren’t empty

Reviewer #2:

===

This box is for your report. All comments included here will be passed to the authors.

===

Please consider the following questions:

- Are the methods appropriate to the aims of the study, are they well described, and are necessary controls included?

- Are the conclusions adequately supported by the data shown?

- Are sufficient details provided to allow replication and comparison with related analyses that may have been performed?

- Is the method likely to be of broad utility? Is any software component easy to install and use? Please indicate briefly the novel features and/or advantages of the method, and/or please reference the relevant publications and which methods, if any, it should be compared with.

- Is the paper of broad interest to others in the field, or of outstanding interest to a broad audience of biologists?

Long read based transcriptome sequencing is now considered as state of the art due to the advantages it offers in full length cDNA/RNA sequence generation and isoform detection. Many tools have been developed in the last 4-5 years to enable the analysis of long read data, however the field is still seeking a tool that will take full advantage of this technological advance.

In this method paper the authors present Mandalorion v4, which is the latest development of their isoform detection tool. They measure the performance of v4 using benchmark data (generated by PacBio, the LRGASP consortium and publicly available data), simulated data, as well as through the use of spike-in internal RNA variant controls (SIRVs). Including the SIRV isoform mix is important to provide an experimental benchmark. An important part of the experimental plan included the analysis with and without an annotation file. This is particularly important in the cases where the transcriptomes of non-model, not well annotated organisms are being studied. Transcripts were also quantified and the performance of Mandalorion v4 was compared to the StringTie tool, a widely used isoform detection tool, initially developed for short read data.

Overall, the experimental design utilizes verifiable benchmark data and reagents, and the methodology is well described.

The initial evaluation in terms of recall and precision tests (with and without annotation) was performed using simulated PacBio iso-seq data and SIRVs which provide a truth dataset. The authors claim comparable performance when the annotation file was used, but a superior performance by v4 compared to StringTie, particularly over complex SIRVs, when the annotation file was not used. Quantification performance was similar between the two tools, with generated transcript TPM values highly correlating to the truth set.

Next the authors assessed the performance of the two tools by analyzing a publicly available reference dataset (also PacBio iso-seq based). Here in the absence of ground truth, the authors focused on whether isoforms matched the GENCODE annotation and whether they reflected the actual read alignments they were based on. Again, the performance of Mandalorion v4 was superior when an annotation file was not provided. Importantly, there was concordance between the isoforms detected with and without annotation.

Overall, the development and benchmarking of the fully packaged Mandalorion v4 tool is a welcome addition to the toolkits required to make full use of long read sequencing approaches.

It shows good performance without an annotation file, is of high interest to the transcriptomics and genome annotation community, - and in the view of this reviewer not only for the analysis of not well annotated genomes, but also in the cases of highly complex cancer transcriptomes.

The analysis toll is now well packaged and more user friendly and should allow broad use by researchers

Major comments

1. The authors need to assess the effect of non-full length (originally degraded RNAs, or incomplete cDNAs synthesized) and cDNA synthesis artifacts on the performance of Mandalorionv4. It is expected that real-world data will contain various proportions of such sequences and this issue could be a limiting factor for the usefulness of the tool.

I think we missed the mark when describing how the data for this study was simulated or generated and have thus created this misunderstanding. All data sets analyzed in this study contain non-full length cDNA sequences. The simulated data were generated with IsoSeqSim simulates errors, including cDNA sequence truncations, at rates that are derived from actual experimental PacBio Iso-Seq data. The settings for simulations were similar to the ones used in the Nature Biotechnology paper evaluating IsoQuant for example. The SIRV data we analyzed was generated for LRGASP using a publicly available protocol that relies on a modified Smart-Seq2 protocol and therefore will also contain truncations based on internal oligodT priming and premature template switching. The same is true for the UHR data. As such this manuscript evaluates Mandalorion (and StringTie, Bambu, and IsoQuant) on representative PacBio Iso-Seq data.

In summary, in this manuscript, the performance of Mandalorion was assessed on

three separate data sets - one simulated, two generated - which all contain truncated

cDNA sequences typical for current PacBio Iso-Seq data.

Mandalorion has optional settings to deal with atypically highly truncated data which adjusts the threshold for filtering isoforms that could be truncations of longer isoforms. By default, these internal isoforms are discarded unless they are more abundant than the isoform that contains them. In a highly truncated dataset this threshold can be adjusted upwards to discard internal isoforms unless they are much more abundant than the isoform that contains them.

Because Mandalorion has this capability, I don’t think we could fairly compare it to other tools on atypically highly truncated datasets. While we can adjust Mandalorion to perform well with these datasets we don’t know how to adjust the other tools.

2. The authors base their comparison on the StringTie tool, however the LIQA tool (Hu et al 2021 in this journal) has claimed a higher accuracy than Mandalorion. It is important to include a comparison with this tool, so that readers can obtain a more comprehensive picture of comparative performance.

While LIQA does quantify isoforms, it does not identify isoforms. That means it relies entirely on a genome annotation like GENCODE as the basis for its quantifications. It is therefore not the same class of tool as Mandalorion, StringTie, Bambu, and IsoQuant, which are all now included in the manuscript.

Also Mandalorion was run on regular ONT data for this 2021 paper which is explicitly not recommended. This might have caused the differences in Pearson’s r-values of 0.992 and 0.6 value determined in the 2021 paper.

Even though LIQA is a different type of tool, it does quantify transcripts and is at least in principle - although with a list of caveats - comparable to the tools in our manuscript. So we installed LIQA and ran it on the ~19 million read simulated mouse data set we used for our comparison, which is much larger than the datasets in the LIQA paper. We timed the speed at which LIQA processed genes and it would have taken the tool weeks to complete its analysis. This makes running LIQA impractical. The authors of the tool seem to have included a multi-threaded option in the LIQA code but didn’t document this feature. When we read their code, we were able to run the tool multithreaded on 60 cores, and it completed in about 3 days. However, it only produced results for about 10,000 isoforms - a fraction of the isoforms in the sample or the annotation. When we performed the most charitable analysis, focusing only on those 10,000 isoforms, LIQA had a Pearson’s r-value of about 0.75 compared to the >0.94 for all tools in our manuscript. In short, LIQA is a different class of tool, impractically slow, its undocumented speed-up is potentially broken, and even the most forgiving analysis sees it dramatically underperforming compared to all tools in this manuscript.

We are not opposed to comparing more tools, in fact, we included both Bambu and IsoQuant in our manuscript now, but those tools are similar in function to Mandalorion and complete their analysis in a reasonable amount of time. Unless the reviewer insists, I do not think including LIQA and our analysis of its performance in the manuscript is a good idea due to the concerns I outlined above.

Minor comments

Although data on isoform read counts are presented (Fig.5B) it is not clear what the read count threshold is (one read?) for calling an isoform and the proportional effect of isoform read counts on recall and accuracy. This needs to be clearly described in the text, as well as a table on saturation, showing raw read numbers required to call the isoforms.

To address concerns by reviewer 1, we included more tools in this analysis which made it necessary to replace figure 5B with figures that allowed easier comparison of more tools. We now simply show what percent of isoforms identified by each tool (using their respective default settings) are support either at the splice junction chain level (SJC) or Splice-junction chain + accurate ends (SJC+E) level by a single read in the entire dataset.

Which version of the SIRVs was used? One can assume that it is set 4, please include in main text.

It is SIRV set 4. We have added this text now

Figure 2: in the distance threshold graphs it is hard to distinguish between datasets and tools used, the lines run to close. Maybe use different colour scheme/or thinner lines.

Because we are now including more tools in the analysis, we dropped the R2C2 and Combined datasets from the manuscript. We did so because we needed to simplify and streamline the analysis. This figure is now much easier to read.

Figure 3: it is hard to work out what is not correct with the false positive isoforms when the content of the box is dense. What would be helpful is to clearly show which SIRV isoforms were detected, and which ones were not detected (some seem to escape) by the two tools.

It is true that these types of genome browser shots can become too busy or zoomed out to allow conclusions on individual isoforms. They are meant more to make general points about the data. To provide conclusive information of which tool detects what isoform, we are now including an Excel file (Table S2) with information on each tool/condition and what SIRVs it identifies correctly, misses, or misclassifies.

Figure 4: please comment in the text on what appears to be an overestimation of TPMs by the tools and why this would be the case.

We have now included text on this. I think it is telling that this overestimation largely disappears without an annotation file. Basically, these tools likely overestimate the expression of annotated but low expressed isoforms in otherwise highly expressed loci.

Fig 5: Please include the genomic coordinates shown for the annotated loci.

We have included the coordinates in the figure legend.

**2^nd^ round**

**Reviewer 1**

The authors have made significant improvements to the manuscript and to Mandalorion v4 itself. I particularly appreciate the expansion of the number of compared methods in the benchmark and the improvements to user friendliness of the software. I have a few small comments following.

1. Can the authors elaborate more on the undercounting of expression TPMs in Mandalorion? I wouldn't expect a systematic undercounting of TPM to be possible given that they must sum to 1 million. Is there a small subset of transcripts getting dramatically over-counted? Are the 'missing' counts going to transcripts that were not included in the 21802 transcripts evaluated, and if so is that a concern?

2. The authors "calculated the percentage of isoform overlap between pairs of tools and conditions as (isoforms shared

between pair/(isoforms unique to sample 1+isoforms unique to sample 2))*100". I find this formula surprising as it could lead to percentages that are higher than 100% as I understand it (perfect overlap would give an infinite value). Perhaps the author's meant to use the Jaccard index instead?

3. I would still like further clarification about the simulated dataset and the definition of TP/FP in that evaluation. Does the simulated data contain expressed, unannotated transcripts, and if so, how many transcripts and with how much abundance? And are expressed, unnanotated transcripts considered the same as the expressed, annotated transcripts when computing TP/FP values?

**Authors’ response**

1. Can the authors elaborate more on the undercounting of expression TPMs in Mandalorion? I wouldn't expect a systematic undercounting of TPM to be possible given that they must sum to 1 million. Is there a small subset of transcripts getting dramatically over-counted? Are the 'missing' counts going to transcripts that were not included in the 21802 transcripts evaluated, and if so is that a concern?

That is a very good question. Mandalorion doesn’t use a complex model to evaluate expression. It simply counts how many reads are uniquely assigned to an isoform during the isoform identification stage and then divides that number by the total number of reads in the input dataset fasta/q file(s). The reason this results in slight undercounting in the simulated data set is that not all reads that were simulated for an isoform will be assigned to that isoform by Mandalorion. Mandalorion filters reads based on their alignments, in particular it filters reads with errors around splice sites (often an indicator of misalignments). Overcounting would stem from Mandalorion uniquely assigning a read to the wrong isoform which it seems is quite rare. In summary, the missing counts stem from filtered reads that are simply discarded. Because of the small size of this effect (MARD of ~0.06) we don’t think this is a major concern. Also, we are hesitant to start modeling this behavior because the correlation of Mandalorion-based quantification to the ground truth is so strong and the current approach is so simple. Further, Iso-Seq and in particular R2C2 reads are also becoming more and more accurate, likely further minimizing this problem going forward.

2. The authors "calculated the percentage of isoform overlap between pairs of tools and conditions as (isoforms shared between pair/(isoforms unique to sample 1+isoforms unique to sample 2))*100". I find this formula surprising as it could lead to percentages that are higher than 100% as I understand it (perfect overlap would give an infinite value). Perhaps the author's meant to use the Jaccard index instead?

We indeed used the Jaccard index. We added that to the text and updated the formula.

Thank you for catching that mistake.

3. I would still like further clarification about the simulated dataset and the definition of TP/FP in that evaluation. Does the simulated data contain expressed, unannotated transcripts, and if so, how many transcripts and with how much abundance? And are expressed, unnanotated transcripts considered the same as the expressed, annotated transcripts when computing TP/FP values?

Apologies for not making this clear. For the isoform identification stage all tools were provided with the standard gencode annotation. The simulated dataset contained artificial transcript isoforms that are not in that standard gencode annotation. The simulated dataset also only contained a subset of the standard gencode annotation. The exact composition (annotated and artificial transcript isoforms) of the simulated dataset was released by the LRGASP consortium. To calculate TP/FP we used that composition which in essence contained all and only True Positive transcript isoforms.

We have added the following text to the result section to make all of this more clear.

“The simulated mouse data contained a subset of annotated isoforms present in the GENCODE genome annotation as well as artificial isoforms not present in the GENCODE genome annotation provided to the tools. The genome annotation - if provided to the tools - therefore contained many annotated transcripts that weren’t simulated and didn’t contain any of the simulated artificial transcripts. To evaluate the performance of each tool on this simulated dataset - with or without provided genome annotation - we compared the isoforms they identified to the ground truth annotation - all isoforms that were actually present in the simulated dataset (annotated or artificial). The real mouse PacBio data contained SIRV (set 4, Lexogen) synthetic spike-in transcript isoforms of known sequence. All SIRV isoforms were present in the genome annotation provided to the tools as part of synthetic gene loci. No additional “decoy” isoforms were present in the annotation either, meaning that tools were provided the ground truth for the SIRV isoforms. To evaluate the performance of each tool on these synthetic transcript isoforms - with or without provided genome annotation - we compared the isoforms they identified to the same ground truth annotation.”
